# Regional microbial content of fermented traditional and industrial East Mediterranean sausages from the islands of Cyprus and Mytilini

**DOI:** 10.20517/mrr.2024.47

**Published:** 2024-10-28

**Authors:** Eleni Kamilari, Catherine Stanton, Dimitrios Tsaltas, R. Paul Ross

**Affiliations:** ^1^APC Microbiome Ireland, University College Cork, Cork T12 YT20, Ireland.; ^2^School of Microbiology, University College Cork, Cork T12 K8AF, Ireland.; ^3^Department of Biosciences, Teagasc Food Research Centre, Moorepark, Fermoy, Co., Cork P61 C996, Ireland.; ^4^Department of Agricultural Sciences, Biotechnology and Food Science, Cyprus University of Technology, Lemesos 3036, Cyprus.

**Keywords:** Sausages, fermented, Mediterranean, 16S rRNA gene, ITS loci, high-throughput sequencing, interaction networks

## Abstract

**Objective:** To characterize the microbial biodiversity of fermented sausages from the East Mediterranean islands of Cyprus and Mytilini, and to identify differences between the microbial diversity of traditionally and industrially produced Cypriot sausages.

**Method:** The microbial diversity of thirty sausages from Cyprus and Mytilini (traditionally and industrially produced) was analyzed using high throughput sequencing (HTS) (amplicon sequencing) of 16S rRNA gene and ITS loci fragments. By applying microbial signature detection and machine learning algorithms, we identified key microbes that distinguish traditionally produced sausages from those produced industrially. Focusing on selected microbial taxa and using interaction network analysis, we identified associations among the sausages’ microbiota that may affect the shaping of the sausages’ microbial consortia.

**Results:** Cypriot and Mytilini sausages indicated increased relative representation of *Lactobacillus* and *Leuconostoc*. Cypriot sausages were distinguished by the presence of the fungi *Debaryomyces hansenii* and *Candida spp.*, whereas Mytilini sausages by the bacteria *Lactococcus* and *Streptococcus*. Traditionally produced sausages from the Pitsilia region of Cyprus were differentiated by the presence of the species *Lactobacillus helveticus*, whereas industrially produced sausages were differentiated by the species *Leuconostoc mesenteroides*. Focusing mainly on *Lactobacillus* and *Leuconostoc*, negative associations with pathogenic bacteria, such as *Salmonella*, and spoilage fungi, such as *Fusarium poae*, were revealed.

**Conclusion:** The present metataxonomic analysis provided insights into the microbial communities that characterize Cypriot and Mytilini sausages. The findings provide an indication that the microbial diversity might be applied as an additional marker of Cypriot sausages’ authenticity.

## INTRODUCTION

Fermented meat products, such as sausages, have been an important part of the gastronomical culture of East Mediterranean countries. Traditional Cypriot sausages are highly favored locally, with the regional Pitsilia sausages recently registered as protected geographical indications (PGI) by the European Commission (EU No: PGI-CY-02369 - 15.9.2017). Their distinctive sensory characteristics arise from their specific processing conditions. Specifically, ground pork is mixed with salt, spices, and herbs such as coriander, and then stuffed into casings made from pork intestines^[1]^. The fermentation process involves soaking the sausages in local, red dry wine for eight days, followed by smoking over wood from indigenous bushes or trees for two to five days. Pitsilia is a mountainous region with a cold climate, which enhances the preservation of meat products. Traditional Mytilini sausages are also made from pork meat, salt, herbs, and spices, all encased in a casing derived from pork intestine. Mytilini sausages are also smoked in beech or other type of wood for seven hours. Their main difference from traditional Cypriot sausages is the absence of red wine. Industrialized sausages can also be found in the Cypriot and Mytilini markets. Following wine’s addition, industrialized sausages are treated with cold smoking, and preservatives such as phosphates and nitrate are added.

The microbial diversity of sausages is influenced by a combination of contributors, including their origin, the type of meat, processing and fermentation conditions, temperature, ripening stage, the addition of additives, salt, herbs, spices, and preservatives, the storage conditions, the use of starter cultures, *etc.*^[2-6]^. The combinatory effect of these factors selects for the development of a unique microbial pattern that affects the sensorial and qualitative characteristics of the final product. In spontaneously fermented sausages, the fermentation initiates with a pool of microbes shaped by microorganisms existing in the meat, in the other added ingredients, and other microbes from the environment^[7]^. Previous studies have shown that pre-fermentation sausages’ microbiota is composed of several unwanted microbes, such as representatives of the families *Lactobacillaceae*, *Bacillaceae*, *Actinomycetaceae*, *Enterobacteriaceae*, and *Pseudomonadaceae*, yeasts, and molds^[8,9]^. Post-fermentation, the microbiota is dominated by coagulase-negative staphylococci and lactic acid bacteria (LAB), including representatives of *Lactobacillaceae*, *Leuconostocaceae*, and *Streptococcaceae*. Additionally, the spore-forming *Bacillaceae*, and spoilage microorganisms, such as *Enterobacteriaceae*, *Pseudomonadaceae*, and *Actinomycetaceae*, can also be found^[10-12]^.

To prevent the growth of pathogenic and spoilage microorganisms, a common practice involves the addition of preservatives, previously synthetic and nowadays natural^[13,14]^. More recently, an alternative practice to control the quality of fermented sausage, recognized by the US Food and Drug Administration (FDA), is called biopreservation^[15]^. Biopreservation involves the administration of Generally Recognized as Safe (G.R.A.S) organisms, such as LAB and/or their metabolites, including bacteriocins, bacteriophages, and bacteriophage-encoded enzymes for monitoring the presence of unwanted microorganisms in food and foodstuff. Additional sausage processing and preservation approaches involve curing, fermentation, ripening, heat treatment, high-pressure processing, marination, smoking, and drying^[16,17]^.

Sausage production conditions create an unfavorable environment for the microbiota. Therefore, only microbes with specific metabolic adaptations may survive^[18]^. Those microbes are members of the regional microbiome, the development of which is influenced by environmental factors, including climate, topography, and soil^[19-21]^. The distinctive microbiome of each sausage type influences the sensory characteristics of the final product through well-orchestrated networks^[22]^. Microbial interactions performed among different taxonomic groups lead to the coexistence of specific, phylogenetically diverse microbiomes. The development of co-occurring and co-exclusionary networks based on the presence/absence and relative abundance of key microbes enables the understanding of the relationships among the members of the community^[23]^. Comparing interaction networks generated from microbial communities developed in sausages produced using different manufacturing conditions and having different origins, may provide insights into understanding the factors that influence the microbial diversity developed in different types of sausages.

The present study aimed to characterize the microbial composition of Cypriot and Mytilini sausages using metataxonomic sequencing, aspiring to enhance the existing knowledge of the microbial consortia that are shaped in traditional and industrial sausages undergoing spontaneous fermentation. It is a follow-up study^[1]^ of characterizing Cyprus sausages’ bacterial communities improved with the addition of more samples and enriched with the characterization of the fungal diversity. Additionally, it provides a snapshot of Mytilini sausages’ microbial communities. Using co-occurring and co-exclusionary networks, the outcome of the study may increase our understanding of the microbial interactions developed in spontaneously fermented sausages. Furthermore, the study provides insights into using the microbiome as an additional tool for fingerprinting traditional sausages for authentication purposes.

## METHODOLOGY

### Sausages collection

Sausage samples were collected between December 2021 and January 2022. Samples included 22 pork sausages from Cyprus and 8 from Mytilini [Table 1]. Sausages that were originated from the same region were produced by different companies. All sausages were prepared without the addition of starter cultures (spontaneous fermentation). Samples were put in cold storage boxes and transferred to University College Cork, Ireland, after about 5 working days. All samples were stored at -20 °C until processing.

**Table 1:** Information about the collected samples

**Sample ID**	**Country**	**Area**	**Traditional/industrial**	**Smoked**	**Wine**	**Preservatives**
C1	Cyprus	Lemesos	Industrial	Yes	Yes	Yes
C2	Cyprus	Lemesos	Industrial	Yes	Yes	Yes
C3	Cyprus	Pitsilia	Traditional	Yes	Yes	No
C4	Cyprus	Pitsilia	Traditional	Yes	Yes	No
C5	Cyprus	Pitsilia	Traditional	Yes	Yes	No
C6	Cyprus	Pitsilia	Traditional	Yes	Yes	No
C7	Cyprus	Nicosia	Industrial	Yes	Yes	No
C8	Cyprus	Nicosia	Industrial	Yes	Yes	No
C9	Cyprus	Nicosia	Industrial	Yes	Yes	No
C10	Cyprus	Nicosia	Industrial	Yes	Yes	No
C11	Cyprus	Nicosia	Industrial	Yes	Yes	No
C12	Cyprus	Nicosia	Industrial	Yes	Yes	No
C13	Cyprus	Nicosia	Industrial	Yes	Yes	No
C14	Cyprus	Nicosia	Industrial	Yes	Yes	No
C15	Cyprus	Pitsilia	Traditional	Yes	Yes	No
C16	Cyprus	Pitsilia	Traditional	Yes	Yes	No
C17	Cyprus	Pitsilia	Traditional	Yes	Yes	No
C18	Cyprus	Nicosia	Industrial	Yes	Yes	Yes
C19	Cyprus	Nicosia	Industrial	No	Yes	Yes
C20	Cyprus	Nicosia	Industrial	No	Yes	Yes
C21	Cyprus	Nicosia	Industrial	No	Yes	Yes
C22	Cyprus	Nicosia	Industrial	No	Yes	Yes
G1	Greece	Mytilini	Industrial	Yes	No	Yes
G2	Greece	Mytilini	Industrial	Yes	No	Yes
G3	Greece	Mytilini	Industrial	Yes	No	Yes
G4	Greece	Mytilini	Traditional	Yes	No	Yes
G5	Greece	Mytilini	Traditional	Yes	No	Yes
G6	Greece	Mytilini	Traditional	Yes	No	Yes
G7	Greece	Mytilini	Traditional	Yes	No	Yes
G8	Greece	Mytilini	Traditional	Yes	No	Yes

### DNA isolation and DNA yield measurement

Sample homogenization was performed by mixing 5 grams of sausages with 45 mL of maximum recovery diluent (MRD) in a Stomacher 400 Circulator (Seward, UK) at a shaking speed of 300 rpm for 2 min. Each homogenized sample in a volume of 1.8 mL was transferred to a 2 mL tube and the cell-free supernatant was separated from the cell pellet by centrifugation at 16,000 × *g* for 5 min at 4 °C. The T cell pellet was diluted in 450 µL Solution MBL (MoBio Laboratories Inc., Carlsbad, CA, USA) and the microbial DNA was extracted according to the protocol instructions of DNeasy® PowerFood® Microbial Kit (MoBio Laboratories Inc., Carlsbad, CA, USA). The isolated DNA was kept at -20 °C until processing.

Extracted DNA yield measurement was performed using the Qubit 4.0 fluorometer (Invitrogen, Carlsbad, CA) and Qubit dsDNA HS Assay Kit (Invitrogen). DNA quality was estimated by calculating the A260/A280 and A260/A230 absorbance ratios in a spectrophotometer (NanoDrop Thermo Scientific, USA).

### Illumina MiSeq barcoded sequencing for 16S rRNA gene in bacteria and ITS region in fungi

For the 16S rRNA region and ITS loci amplification, the library preparation and sequencing were executed in an Illumina MiSeq platform as previously described^[24-26]^.

### Data interpretation

Raw fastq sequences filtering, and alpha and beta diversity analyses were executed using Qiime 2 version 2021.11, as previously described^[27]^. Samples were rarefied in 17784 for bacteria and 16957 for fungi. Statistically significant differences among sausages from different areas or manufacturing conditions based on their beta diversity were assessed using non-parametric permutational analysis of variance (PERMANOVA)^[28]^, as described by Kamilari *et al.*^[25]^. Taxonomy assignment for 16S rRNA gene sequences and ITS regions into OTUs was based on the q2-feature-classifier^[29]^ against the Greengenes 13_8 99% OTUs reference sequences^[30]^ and the UNITE ITS database (9_12 release)^[31]^, respectively. Sequence filtering was conducted to filter out incomplete taxonomies that could not be detected at the order level. The identification of microbial signatures was performed using the LEfSe algorithm^[25,32]^ and the random forest algorithm in Qiime2, as described previously^[33-35]^. Statistically significant correlations between area/processing conditions and identified microbiota were estimated using CoNet^[36]^, and visualized using Cytoscape 3.2.1^[37]^, as described by Kamilari *et al.*^[35]^.

The raw sequence data were archived in the NCBI sequence read archive (SRA) with BioProject PRJNA 1010532.

## RESULTS

### Microbial population and diversity metrics in sausages

Sausages originating from Cyprus (Nicosia, Lemesos, Pitsilia) and Mytilini were separately examined for their microbial diversity. The 16S rRNA amplicon sequencing results showed that the 30 specimens generated an average of 37,851.47 sequencing reads (range = 19,570-107,301, STD = 15,275.43, Supplementary Table 1), and 155.97 OTUs (range = 32-696, SD = 137.37; Supplementary Table 1) per sample. The ITS loci amplicon sequencing results from 23 samples produced an average of 47,687 sequencing reads (range = 23,571-81,687, STD = 13,842; Supplementary Table 2), and 202 OTUs (range = 37-327, SD = 87; Supplementary Table 2) per specimen. Seven samples were excluded from the analysis owing to a reduced number of reads (< 8,000).

Initially, the Shannon, Simpson, and Chao1 indices were analyzed to estimate the alpha diversity of the bacterial and fungal communities [Supplementary Tables 1 and 2]. The results indicated that there was no significant difference among the areas of sausage production, or the applied manufacturing conditions, using the Kruskal–Wallis test [Supplementary Table 3, Shannon index].

### Regional microbial beta diversity

To assess the existence of unique microbial signatures among sausages produced in different areas or between traditionally and industrially produced Cypriot sausages, we calculated the beta diversity based on the weighted and unweighted UniFrac distances [Supplementary Table 4]. No notable difference was observed in microbial diversity among sausages produced in different areas, or between traditionally and industrially produced sausages, based on the PERMANOVA test.

### Microbial community taxonomic profile

**Figure 1:**
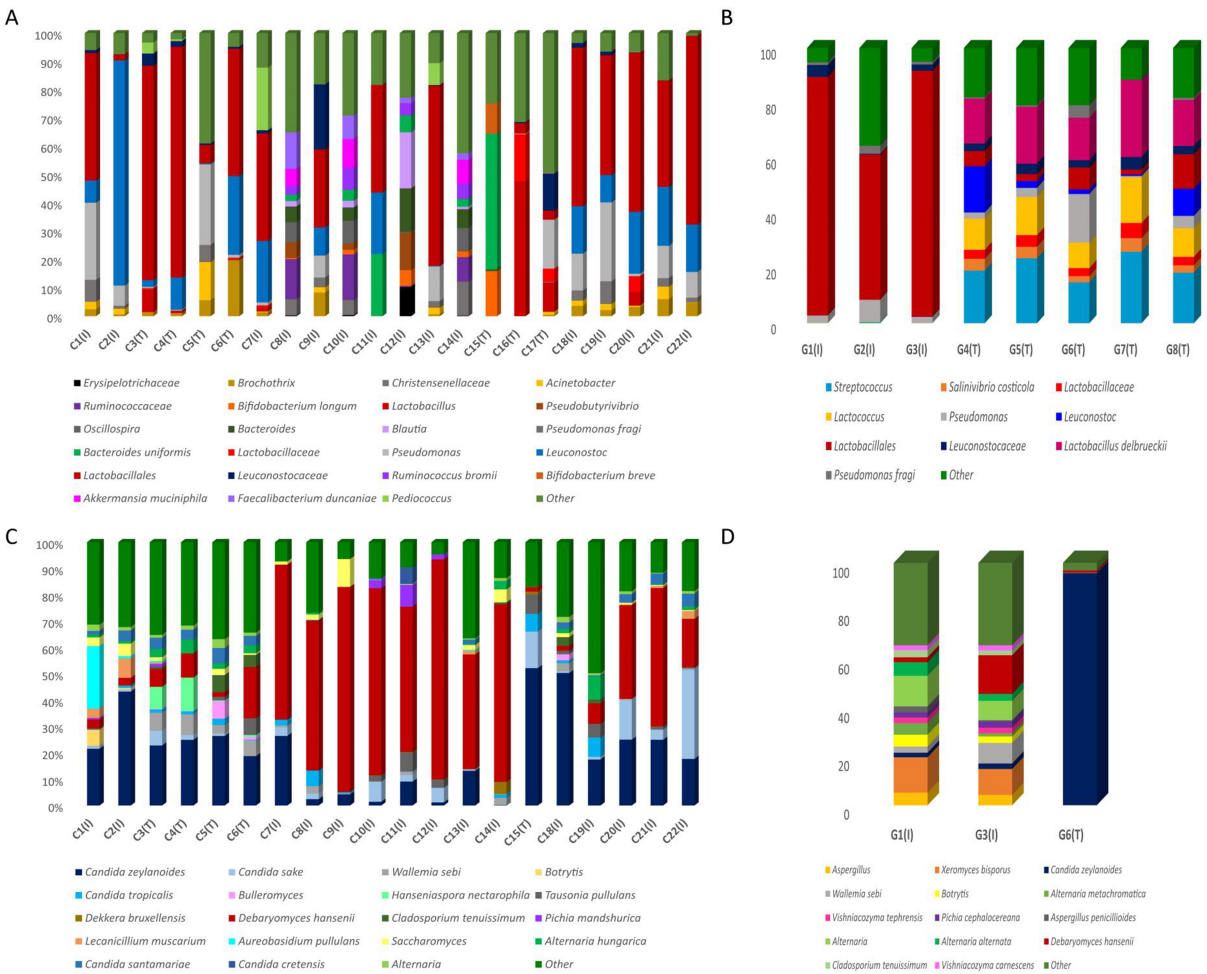
The relative abundance of the most abundant microbes identified at the species level (mostly including the family and the genus level) based on 16S rRNA gene sequencing for (A) Cyprus, (B) Mytilini, and based on *ITS* gene loci sequencing for (C) Cyprus, (D) Mytilini. The code (T) refers to traditional sausages, whereas the code (I) refers to industrial sausages.

The predominant bacterial genus in Cypriot and Mytilini sausages was *Lactobacillus* [Figure 1A and B]. Some Cypriot sausages were defined by a higher relative abundance of *Leuconostoc* (7%-27%). Additionally, *Pseudomonas* and *Brochothrix* were detected in lower relative abundances in some sausages (0%-7% and 0%-8%, respectively). Industrially produced sausages from Mytilini were dominated by the presence of *Lactobacillales* (52%-89%), whereas traditionally produced were dominated by *Lactobacillus delbrueckii* (15%-28%), *Lactococcus* (9%-16%), and *Streptococcus* (15%-26%). The genera *Leuconostoc* (0%-16%), *Salinivibrio* (0%-4%), and *Pseudomonas* (0%-17%) were detected in lower relative abundances in traditionally produced Mytilini sausages. Regarding the fungal diversity, Cypriot sausages exhibited an enhanced representation of the species *Debaryomyces hansenii* (0.1%-83%), *Candida zeylanoides* (0.2%-52%), and *Candida sake* (0%-32%) [Figure 1C]. Reduced relative abundances were also observed for members of the genera *Saccharomyces* (0%-19%) and *Alternaria* (0%-4%), the species *Aspergillus penicillioides* (0%-5%), and *Wallemia sebi* (0%-9%). Most Mytilini sausages were excluded from subsequent analyses due to insufficient sequencing output (< 10,000 reads cutoff after filtering). One Mytilini sausage was dominated by the species *C. zeylanoides* (96%) [Figure 1D]. The other two showed an enhanced representation of the species *Xeromyces bisporus* (10%-14%) and members of the genus *Alternaria* (8%-12%). *D. hansenii* was among the dominant species of one sausage (16%). Other species that were detected in lower relative abundances included *Wallemia sebi* (0.2%-8%), *Botrytis sp.* (0%-4%), *Vishniacozyma tephrensis* (0%-2%)*, Vishniacozyma carnescens* (0%-2%)*, Pichia cephalocereana* (0%-3%)*,* and *Cladosporium tenuissimum* (0%-3%).

### Identification of microbial biomarkers

**Figure 2:**
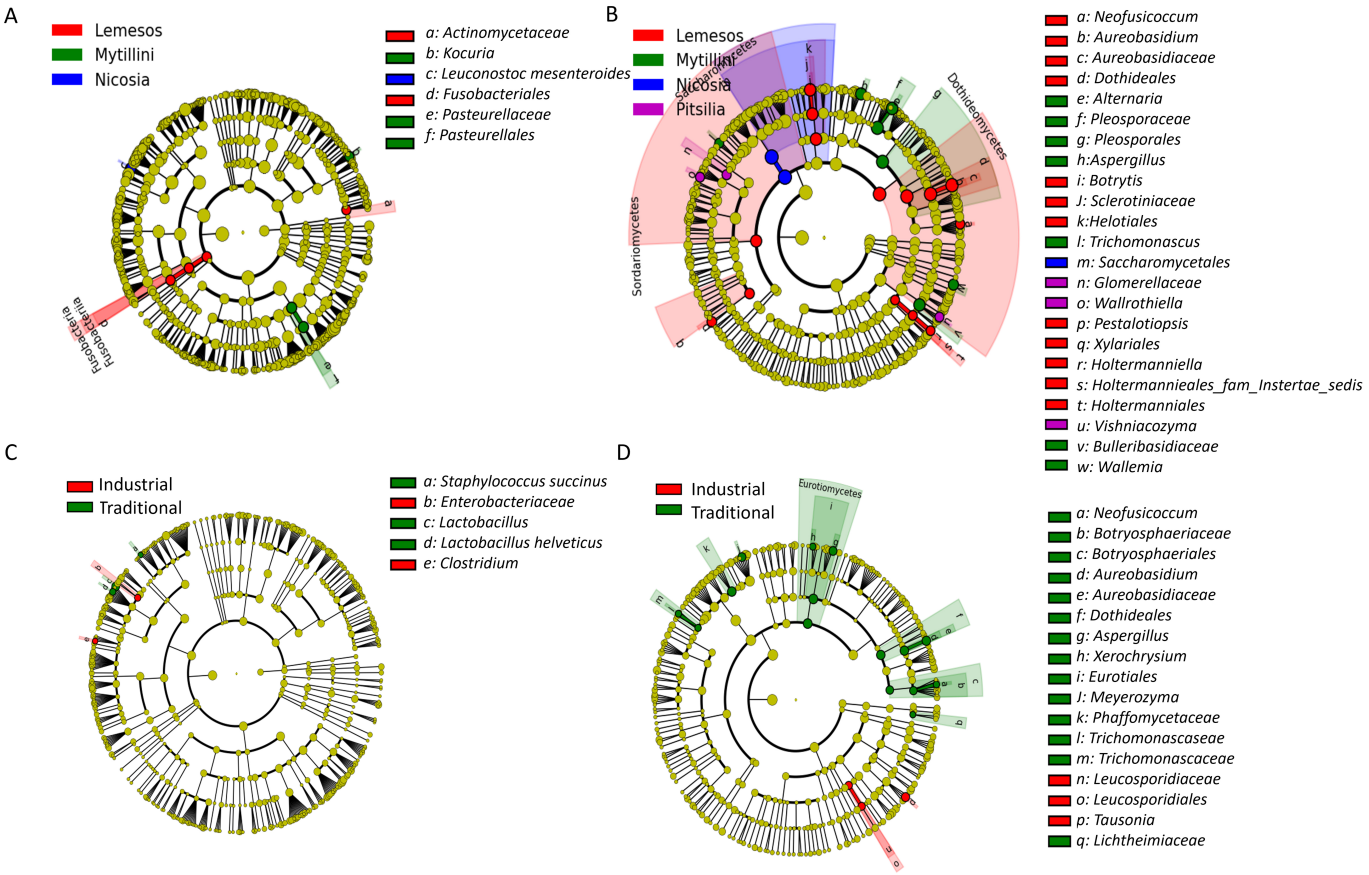
Microbial signatures discovery using the LEfSe algorithm, showing (A) bacterial taxa with statistically significant over-representation among different areas; (B) fungal taxa with statistically significant over-representation among different areas; (C) bacterial taxa with statistically significant over-representation between traditionally and industrially produced sausages; (D) fungal taxa with statistically significant over-representation between traditionally and industrially produced sausages, based on a non-parametric factorial KW sum–rank test, an (unpaired) Wilcoxon rank–sum test, and LDA. The phylogenetic trees map the taxonomic variances of the identified microbial signatures from class (external part of the circle) to species level (internal part of the circle), combined with a list of taxa with significantly increased representation in different sausage groups. KW: Kruskal–Wallis; LDA: linear discriminant analysis.

The LEfSe tool was used to determine if the relative representation of the detected microbial taxa was disproportionately distributed among sausages produced in different areas where the LEfSe algorithm was applied [Figure 2A and B]. Sausages from Mytilini were distinguished by a greater representation of the bacteria *Kocuria* and the family *Pasteurellaceae*, as well as the undesirable fungi *Alternaria*, *Pleosporaceae*, *Aspergillus*, *Trichomonascus*, *Bulleribasidiaceae*, and *Wallemia*. Cypriot sausages from Nicosia were overrepresented by the species *Leuconostoc mesenteroides*, whereas those from Limassol by members of the bacterial family *Actinomycetaceae* and by the fungal genera *Neofusicoccum*, *Aureobasidium*, *Botrytis*, *Pestalotiopsis*, and *Holtermanniella*. Finally, traditional sausages from Pitsilia indicated an elevated relative abundance of the fungi *Glomerellaceae*, *Wallrothiella*, and *Vishniacozyma*.

The traditional manufacturing conditions applied in Cypriot Pitsilia sausages contributed to a significantly higher relative representation of *Lactobacillus*, including *L. helveticus* [Figure 2C]. The species *Staphylococcus succinus*, and the genera *Neofusicoccum*, *Botryosphaeriaceae*, *Aureobasidium*, *Aspergillus*, *Xerochrysium*, and *Meyerozyma* were also overrepresented [Figure 2C and D]. Industrially produced sausages exhibited an increased relative abundance of *Clostridium* and *Enterobacteriaceae*, and the fungi *Leucosporidiaceae* and *Tausonia*.

### Differentiation of sausages based on origin and manufacturing conditions using random forest algorithm

**Figure 3:**
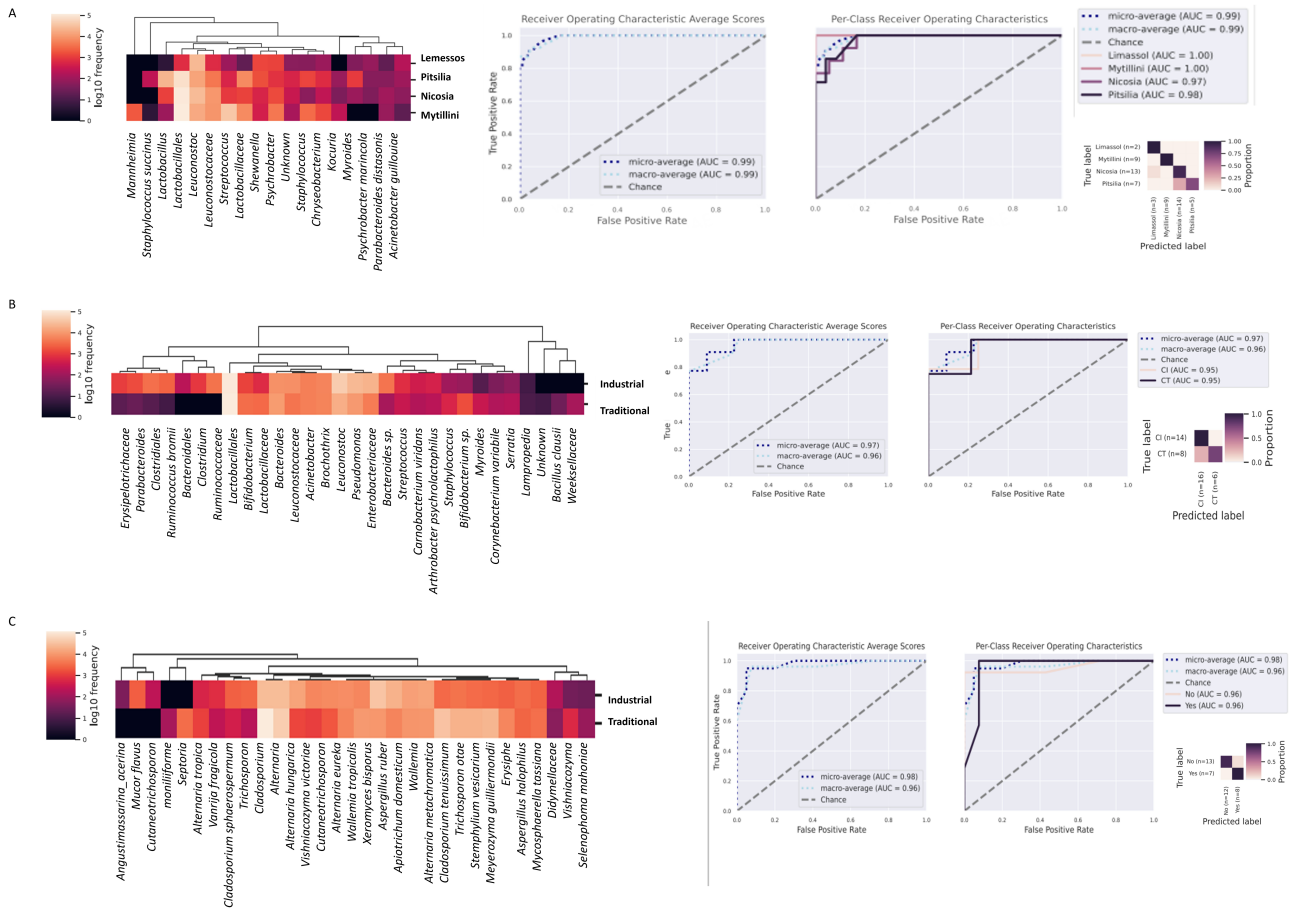
Microbial taxa that can accurately differentiate (A) the bacterial diversity of sausages from different areas; (B) the bacterial diversity of Cypriot traditionally produced and industrially produced sausages; (C) the fungal diversity of Cypriot traditionally produced and industrially produced sausages. Heatmap shows the top microbial predictive features that differentiate sausages per each category. ROC and PCROC analyses for microbial taxa, indicating true and false positive rates for sausages from different areas, industrially or traditionally produced Cypriot sausages, show perfect predictive accuracy (AUC = 0.99, AUC 0.97, respectively). ROC: Receiver operating characteristics ; PCROC: per class receiver operating characteristics AUC: area under the curve.

The random forest algorithm was trained to predict, based on the 16S rRNA gene abundance data, the different origins of the analyzed sausages. The results revealed that sausages from different areas could be separated with reliable predictive capability [area under the curve (AUC) = 0.99 regarding bacteria (Figure 3A)]. This indicates that the algorithm could predict different sausages’ origins with great reliability. The key predictive features that could differentiate sausages of different origins were similar to the microbial signatures that were identified using the LefSe algorithm. Specifically, the taxa *Lactobacillus* and *S. succinus* characterized traditional Pitsilia sausages, and *Leuconostoc* was typical of sausages from Nicosia. However, Mytilini sausages were mostly characterized by the genera *Streptococcus* and *Psychrobacter*.

Cypriot traditional sausages were separated from Cypriot industrial sausages with high predictive accuracy (average AUC = 0.97 for both bacteria and fungi, Figure 3B and C, respectively). Industrial sausages were differentiated by the elevated presence of the bacterial taxa *Erysipelotrichaceae*, *Parabacteroides*, *Clostridium*, *Ruminococcus*, *Leuconostoc*, *Acinetobacter*, *Brochothrix*, *Pseudomonas*, and *Enterobacteriaceae*, whereas traditional sausages by the family *Lactobacillaceae*. Regarding the fungal diversity, industrial sausages were distinguished by increased relative representation of *Mucor flavus*, *Trichosporon*, *Cladosporium*, and *Aspergillus ruber*, whereas traditional sausages by the species *Alternaria tropica*, *Cladosporium tenuissimum*, *Trichosporon otae*, *Stemphylium vesicarium*, and *Meyerozyma guilliermondii*.

### Microbial interaction investigation

**Figure 4:**
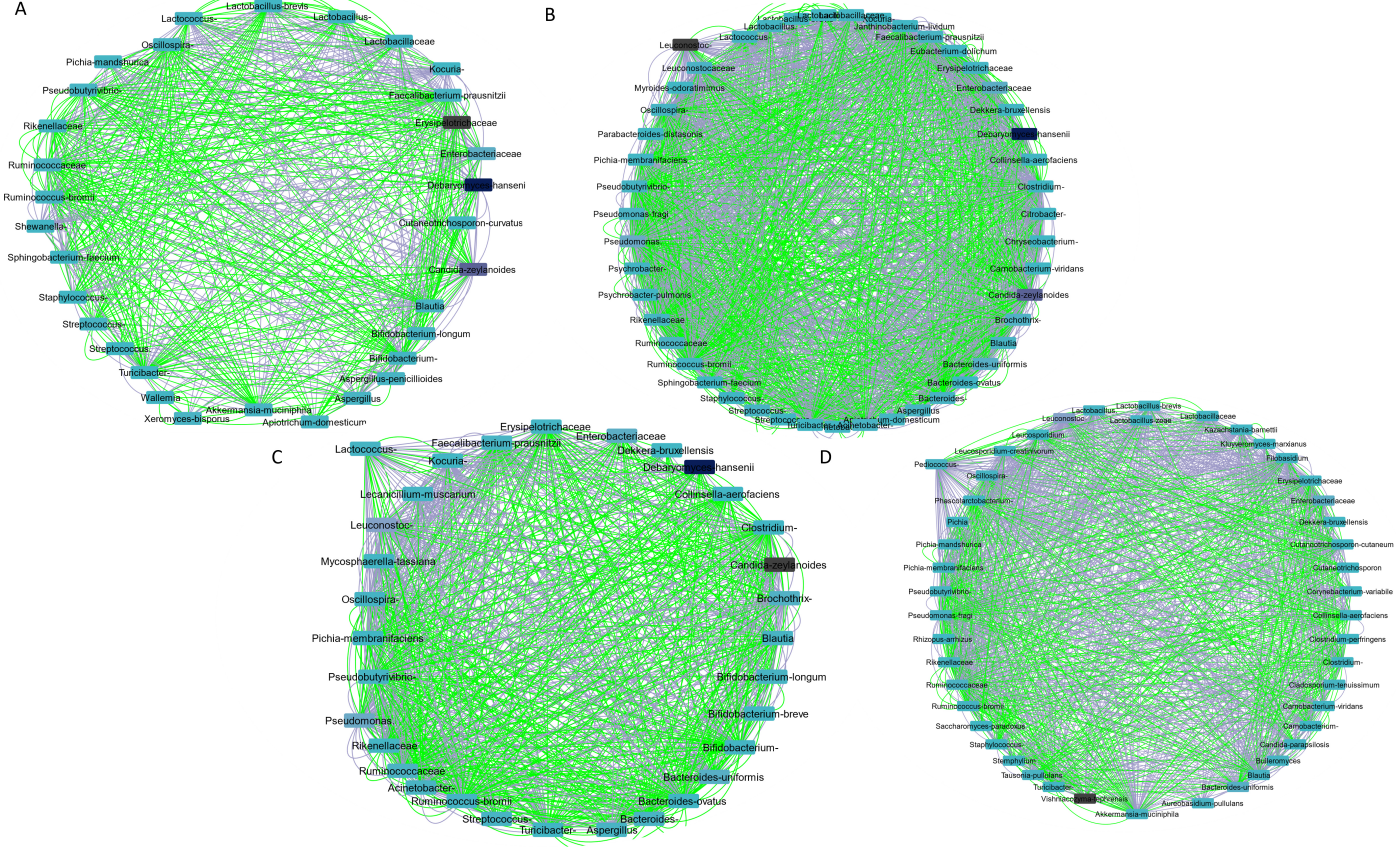
The correlation pattern of microbial communities focusing on (A) *Lactobacillus*; (B) *Leuconostoc*; (C) *Lactococcus*; and (D) *Pediococcus*. Green and purple edges represent positive or negative correlations between two nodes, respectively, based on Spearman’s rank correlation, the Pearson correlation, and the Bray Curtis and Kullback–Leibler dissimilarity matrices. The degree of relative abundance ranges from light to dark blue based on the lower to higher abundant taxa.

To increase our understanding regarding the complicated correlations among the detected taxa, a co-occurrence network was generated using the CoNet algorithm in Cytoscape [Figure 4]. The interactions were chiefly oriented toward the keystone taxa that were overrepresented in Cypriot and/or Mytilini sausages.

Focusing on *Lactobacillus*, one of the most dominant genera in Cypriot and Mytilini sausages and a biomarker of traditional Pitsilia sausages, a positive association was observed with *Streptococcus*, *Lactococcus*, and *Staphylococcus* [Figure 4A]. Negative associations were observed with the taxa *Enterobacteriaceae*, *Pseudobuturivibrio*, *Rikenellaceae*, *Ruminococcaceae*, *Aspergillus*, *Candida zeylanoides*, *Debaryomyces hansenii*, *Pichia mandshurica*, and *Xeromyces bisporus*. Both *Leuconostoc*, a genus that characterized industrial sausages, and *Lactococcus*, which characterized sausages from Mytilini, indicated co-presence with the taxa *Pseudomonas*, *Acinetobacter*, *Candida zeylanoides*, and *Brochothrix*, whereas mutual exclusion was observed for *Clostridium*, *Erysipelotrichaceae*, *Ruminococcacea*, *Debaryomyces hansenii*, and *Pichia membranifaciens* [Figure 4B and C, respectively]. Additionally, *Leuconostoc* indicated negative associations, whereas *Lactococcus* showed positive associations with *Streptococcus* and *Staphylococcus*. *Pediococcus*, a genus that was detected in some Cypriot sausages, indicated positive associations with *Lactobacillus* spp., *Leuconostoc* spp., and *Staphylococcus*, and was negatively associated with taxa, such as *Enterobacteriaceae*, *Erysipelotrichaceae*, *Clostridium*, including *C. perfringens*, *Pseudomonas* spp., *Pseudobutyrivibrio*, *Pichia* spp., *Stemphylium*, *Kluyveromyces marxianus*, and *Kazachstania barnettii* [Figure 4D].

## DISCUSSION

Emerging evidence highlights the presence of identifiable microbial signatures in fermented products of different geographic origins and manufactured using different processing conditions^[5,20,21,38,39]^. This work analyzed the microbial diversity of fermented eastern Mediterranean sausages from the islands of Cyprus and Mytilini using metataxonomic sequencing. Apart from characterizing their microbial diversity, the goal of the study was to identify key microbes that would distinguish between traditionally and industrially produced Cypriot sausages. Furthermore, focusing on dominant taxa, the study aimed to reveal interactions that may affect the dynamics of the microbial community composition in sausages, thereby reflecting sausages’ qualitative characteristics. The study identified distinct microbial signatures in sausages produced in different geographic areas with different manufacturing conditions (Cypriot traditionally and industrially produced) using the Lefse biomarkers discovery and random forest algorithms. Specifically, the random forest algorithm accurately determined the origin and different producing conditions of sausages according to their microbiota. Both algorithms identified the taxa *Lactobacillus* and *S. succinus* as microbial signatures of traditional Pitsilia sausages and *Leuconostoc* of Nicosia sausages. In agreement, Kamilari *et al.* identified *Lactobacillus* and *Leuconostoc* as the dominant taxa in Cypriot sausages and indicated that *Leuconostoc* was mostly associated with industrial, compared to traditional Pitsilia sausages^[1]^. A greater representation of *Lactobacillus* species in Mediterranean fermented meat products, compared to those from Southern Europe, was detected by Van Reckem *et al.*^[4]^. Furthermore, an elevated relative abundance of *Lactobacillus* species was detected in Italian and Spanish spontaneously fermented sausages^[22,38,40]^. Lactobacilli are among the most desired microbes to guide the fermentation process^[41]^. Their capability to ferment sugars and produce lactic acid creates an acidic environment essential for fibrillar protein coagulation. This process favors the stability and cohesiveness of sausages, facilitating slicing^[42]^. Additionally, lactobacilli affect the sensory characteristics of sausages by metabolizing acetic acid, succinic acid, and formic acid^[43]^. Importantly, they are considered gatekeepers against the growth of pathogenic and spoilage bacteria due to the formation of organic and other antimicrobial compounds, such as diacetyl, hydrogen peroxide, and bacteriocins^[44]^. For instance, bacteriocins such as plantaricin (from *Lactiplantibacillus plantarum*), or Sakacin (from *Latilactobacillus sakei*) have been identified to possess biopreservation properties^[45,46]^.

Apart from *Lactobacillus* and *Leuconostoc*, *Lactococcus* was detected in high relative abundances in traditional Mytilini sausages. These bacteria can produce an increased amount of organic acids, including lactic and acetic, throughout their exponential growth, which, in combination with a conversion to ketone and alcohols, contribute to the richer sensory characteristics of the produced sausages^[47,48]^. Importantly, species from these genera are applied as starters for the food industry, owing to their capacity to generate antimicrobial peptides that can inhibit the growth of spoilage and pathogenic microbes. According to online bacteriocin databases Bactibase, Labiocin, and Bagel4, *Lactococcus* has been reported to produce 40 different bacteriocins (http://bactibase.hammamilab.org/main.php; https://labiocin.univ-lille.fr/; http://bagel4.molgenrug.nl/), such as nisin, lacticin, lactococcin G, *etc.*

*Pediococcus* sp. was also detected in several Cypriot sausages. The presence of species of this genus, such as *P. acidilactici* and *P. pentosaceus* is considered beneficial since some strains are classified as probiotic^[49]^. Therefore, some strains have been applied as starter cultures in fermented sausages to guide fermentation process and acidification degree^[50]^. In addition, specific strains possess antimicrobial activity against pathogens due to the formation of pediocin, a bacteriocin with potent antilisterial activity^[51]^. *Pediococcus* sp. has been identified to create negative associations with molds, such as *Stemphylium*, *Kluyveromyces marxianus*, and *Kazachstania barnettii*. Ilavenil *et al.* indicated that a *Pediococcus* strain isolated from Italian ryegrass, *P. pentosaceus* KCC-23, showed effective antifungal activity against species such as *Botrytis* sp. and *Fusarium* sp.^[52]^. Additionally, Fugaban *et al.* isolated two *Pediococcus* species, *P. acidilactici* and *P. pentosaceus*, with inhibitory activity against several mold species, including *Alternaria alternate*^[53]^. In our analysis, we revealed a negative association of *Pediococcus* with spoilage bacteria, such as *Enterobacteriaceae*, *Erysipelotrichaceae*, *Clostridium*, including *C. perfringens*, and *Pseudomonas* spp., members of which are considered foodborne and or opportunistic pathogens^[54]^.

Members of the genus *Clostridium* have been detected in industrially produced sausages. Members of the genus *Clostridium*, such as *C. perfringens*, *C. botulinum*, and *C. difficile*, are considered important foodborne pathogens^[55-57]^. These species produce heat-resistant spores, which can germinate and proliferate in sausages, producing enterotoxins. The presence of these enterotoxins may lead to gastrointestinal symptoms, such as diarrhea, or even death^[55-57]^. Apart from Clostridia, industrially produced sausages indicate the existence of members of the family *Enterobacteriaceae*. Their occurrence in sausages is considered undesirable, due to the production of unwanted flavors and chemical compounds that lead to the deterioration or spoilage of the product^[58]^. Still, they are commonly detected in fresh and frozen meat products^[59,60]^. The presence of Clostridia and *Enterobacteriaceae* in industrially produced sausages indicates the need for improvement in the hygiene and quality of the products.

Coagulase-negative staphylococci, such as *S. succinus*, are considered members of the human and animal skin microbiota, and their presence is associated with dry fermented sausages^[61]^. Some strains can perform proteolysis and lipolysis, contributing to improved sensory characteristics in sausages^[62]^. Therefore, *S. succinus* presence in traditional Pitsilia sausages may contribute to the flavor and taste of the final product. Strains that lack the production of staphylococcal enterotoxins and transferable antibiotic resistance genes are commercially available as starters in fermented sausages^[63]^. However, their presence in human clinical specimens prevents this species from obtaining a qualified presumption of safety (QPS) status by the European Food Safety Authority^[64]^. *Brochothrix* was among the bacterial species that were detected in low relative abundances in most Cypriot sausages. Although *Brochothrix* spp. are non-pathogenic to humans, they are considered of great importance for the meat products industry due to their role in causing premature spoilage in refrigerated products^[65]^. Notably, objectionable odors are produced when oxygen concentrations exceed 0.2%. These odors are mostly detected in dry fermented sausages and are attributed to contamination during packaging operations, as *Brochothrix* spp. do not survive thermal processing but can be reintroduced during packaging.

The predominant fungal species of East Mediterranean sausages was *D. hansenii*. The existence of *D. hansenii* is common in fermented sausages and has been found to influence the dynamics of microbial communities during the ripening of dry sausages in China^[66]^. Additionally, its proteolytic and lipolytic activities positively affect the sensory characteristics of sausages^[67-69]^. This species possesses a particular biotechnological importance for the meat products industry due to its nutritional and probiotic effects, in combination with its increased biotechnological ability^[67,70,71]^. However, recent studies that correlate *Debaryomyces* with inflammation of the gut mucosa and Crohn’s disease create doubts about its addition as a starter in sausages^[72]^.

A common characteristic of Cypriot sausages is the addition of wine. Specifically, in Pitsilia sausages, the fermentation is performed in local red wine for up to 8 days, followed by smoking. This process might favor the growth of *Saccharomyces*. *Saccharomyces* species are common drivers of alcoholic fermentation during vinification^[20]^. Their presence in fermented foods is considered beneficial, not only for improving the sensory characteristics of the product but also because of their antagonistic activity against undesirable microbes. Their metabolic capability to assimilate sugars in grapes to produce ethanol forms a dynamic stress environment for other members of the microbiota. Furthermore, their ability to secrete antimicrobial and antifungal compounds, such as myosin, and other killer toxins, further affects the dynamics of the product’s microbiota^[73,74]^. Alarcón *et al.* indicated that the addition of inactive dry *Saccharomyces cerevisiae* in “salchichón” sausages as an alternative to sodium ascorbate contributed to increased antioxidant activity, a steadier phenolic content, and a reduction in protein and lipid oxidation^[75]^.

The present study is restricted to only twenty-two samples from Cyprus and eight from Mytilini, which is a limiting factor in evaluating the selection of microbial biomarkers associated with origin and manufacturing conditions. Additionally, the geographical distribution of samples should be increased. Sampling should be conducted across various regions and islands around the Mediterranean to identify the microbial signatures associated with each region. Moreover, the season of sampling might influence the composition of microbial communities; therefore, sampling during different seasons will indicate whether microbial diversity remains consistent. While short-read amplicon 16S rRNA gene sequencing is the primary approach used for microbiome studies, it cannot differentiate bacteria at the species level. As indicated from our analysis, bacteria profiling was mostly identified at the genus level, whereas the ITS loci amplicon sequencing could distinguish fungal species. In our future experiments, we will consider applying shotgun sequencing as a more suitable strategy to address this issue. Furthermore, for accurate identification of the microbial population, incorporating blank negative controls during DNA extraction and sequencing may prevent false identification of microbial taxa as members of the population due to DNA contamination from reagents or cross-contamination among samples during sample processing.

In conclusion, the present metataxonomic analysis provided insights into the microbial communities that characterize East Mediterranean sausages from the islands of Cyprus and Mytilini, highlighting key microbial taxa that may distinguish traditionally produced sausages from their industrial counterparts. Understanding the interaction networks among the sausage microbiota members may enhance our comprehension of the complicated interactions that affect the microbial community composition. This study could be incorporated with predictive functional analysis to assess how bacterial and fungal metabolic pathways impact the sensory characteristics of sausages, such as their aromatic profile. Moreover, it may integrate SNIF-NMR, IRMS and inductively coupled plasma atomic emission spectroscopy (ICP-AES)^[76]^, as well as DNA fingerprint characterization studies. Combining our analysis with these approaches may facilitate distinguishing authentic traditional sausages from counterfeit products in the market and offer a fingerprint for combining authentic PGI sausages.

## DECLARATIONS

### Availability of data and materials

Data are available at: https://dataview.ncbi.nlm.nih.gov/object/PRJNA1010021.

### Copyright

© The Author(s) 2024.

## References

[1] Kamilari E, Efthymiou M, Anagnostopoulos DA, Tsaltas D (2021). Cyprus sausages’ bacterial community identification through metataxonomic sequencing: evaluation of the impact of different DNA extraction protocols on the sausages’ microbial diversity representation. Front Microbiol.

[2] Carballo J (2021). Sausages: nutrition, safety, processing and quality improvement. Foods.

[3] Škrlep M, Čandek-Potokar M, Batorek-Lukač N, Tomažin U, Flores M (2019). Aromatic profile, physicochemical and sensory traits of dry-fermented sausages produced without nitrites using pork from Krškopolje pig reared in organic and conventional husbandry. Animals.

[4] (2019). Van Reckem E, Geeraerts W, Charmpi C, Van der Veken D, De Vuyst L, Leroy F. Exploring the link between the geographical origin of European fermented foods and the diversity of their bacterial communities: the case of fermented meats. Front Microbiol.

[5] Leroy F, Geyzen A, Janssens M, De Vuyst L, Scholliers P (2013). Meat fermentation at the crossroads of innovation and tradition: a historical outlook. Trends Food Sci Tech.

[6] Gaydos NJ, Cutter CN, Campbell JA (2016). Fate of pathogenic bacteria associated with production of pickled sausage by using a cold fill process. J Food Prot.

[7] Nychas GJ, Skandamis PN, Tassou CC, Koutsoumanis KP (2008). Meat spoilage during distribution. Meat Sci.

[8] Francesca N, Sannino C, Moschetti G, Settanni L (2013). Microbial characterisation of fermented meat products from the Sicilian swine breed “Suino Nero Dei Nebrodi”. Ann Microbiol.

[9] Hultman J, Rahkila R, Ali J, Rousu J, Björkroth KJ (2015). Meat processing plant microbiome and contamination patterns of cold-tolerant bacteria causing food safety and spoilage risks in the manufacture of vacuum-packaged cooked sausages. Appl Environ Microbiol.

[10] Benson AK, David JR, Gilbreth SE (2014). Microbial successions are associated with changes in chemical profiles of a model refrigerated fresh pork sausage during an 80-day shelf life study. Appl Environ Microbiol.

[11] Połka J, Rebecchi A, Pisacane V, Morelli L, Puglisi E (2015). Bacterial diversity in typical Italian salami at different ripening stages as revealed by high-throughput sequencing of 16S rRNA amplicons. Food Microbiol.

[12] Fontana C, Bassi D, López C (2016). Microbial ecology involved in the ripening of naturally fermented llama meat sausages. A focus on lactobacilli diversity. Int J Food Microbiol.

[13] Pateiro M, Munekata PES, Sant’Ana AS, Domínguez R, Rodríguez-Lázaro D, Lorenzo JM (2021). Application of essential oils as antimicrobial agents against spoilage and pathogenic microorganisms in meat products. Int J Food Microbiol.

[14] Alirezalu K, Pateiro M, Yaghoubi M, Alirezalu A, Peighambardoust SH, Lorenzo JM (2020). Phytochemical constituents, advanced extraction technologies and techno-functional properties of selected Mediterranean plants for use in meat products. A comprehensive review. Trend Food Sci Tech.

[15] Singh VP (2018). Recent approaches in food bio-preservation - a review. Open Vet J.

[16] Gómez I, Janardhanan R, Ibañez FC, Beriain MJ (2020). The effects of processing and preservation technologies on meat quality: sensory and nutritional aspects. Foods.

[17] Feng C, Sun D (2014). Optimisation of immersion vacuum cooling operation and quality of Irish cooked sausages by using response surface methodology. Int J Food Sci Tech.

[18] Ferrocino I, Bellio A, Giordano M (2018). Shotgun metagenomics and volatilome profile of the microbiota of fermented sausages. Appl Environ Microbiol.

[19] Bokulich NA, Thorngate JH, Richardson PM, Mills DA (2014). Microbial biogeography of wine grapes is conditioned by cultivar, vintage, and climate. Proc Natl Acad Sci U S A.

[20] Anagnostopoulos DA, Kamilari E, Tsaltas D

[21] Kamilari E, Tomazou M, Antoniades A, Tsaltas D (2019). High throughput sequencing technologies as a new toolbox for deep analysis, characterization and potentially authentication of protection designation of origin cheeses?. Int J Food Sci.

[22] Franciosa I, Ferrocino I, Giordano M, Mounier J, Rantsiou K, Cocolin L (2021). Specific metagenomic asset drives the spontaneous fermentation of Italian sausages. Food Res Int.

[23] Matchado MS, Lauber M, Reitmeier S (2021). Network analysis methods for studying microbial communities: a mini review. Comput Struct Biotechnol J.

[24] Kamilari E, Anagnostopoulos DA, Papademas P, Kamilaris A, Tsaltas D (2020). Characterizing Halloumi cheese’s bacterial communities through metagenomic analysis. LWT.

[25] Kamilari E, Mina M, Karallis C, Tsaltas D (2021). Metataxonomic analysis of grape microbiota during wine fermentation reveals the distinction of Cyprus regional terroirs. Front Microbiol.

[26] Papademas P, Kamilari E, Aspri M (2021). Investigation of donkey milk bacterial diversity by 16S rDNA high-throughput sequencing on a Cyprus donkey farm. J Dairy Sci.

[27] Kamilari E, Anagnostopoulos DA, Papademas P, Efthymiou M, Tretiak S, Tsaltas D (2020). Snapshot of Cyprus raw goat milk bacterial diversity via 16S rDNA high-throughput sequencing; impact of cold storage conditions. Fermentation.

[28] Anderson MJ (2001). A new method for non-parametric multivariate analysis of variance. Austral Ecol.

[29] Bokulich NA, Kaehler BD, Rideout JR (2018). Optimizing taxonomic classification of marker-gene amplicon sequences with QIIME 2’s q2-feature-classifier plugin. Microbiome.

[30] McDonald D, Price MN, Goodrich J (2012). An improved Greengenes taxonomy with explicit ranks for ecological and evolutionary analyses of bacteria and archaea. ISME J.

[31] Abarenkov K, Henrik Nilsson R, Larsson KH (2010). The UNITE database for molecular identification of fungi - recent updates and future perspectives. New Phytol.

[32] Segata N, Abubucker S, Goll J (2011). Microbial community function and biomarker discovery in the human microbiome. Genome Biol.

[33] Pedregosa F, Varoquaux G, Gramfort A https://www.jmlr.org/papers/volume12/pedregosa11a/pedregosa11a.pdf.

[34] Bokulich NA, Dillon MR, Bolyen E, Kaehler BD, Huttley GA, Caporaso JG (2018). q2-sample-classifier: machine-learning tools for microbiome classification and regression. J Open Res Softw.

[35] Kamilari E, Tsaltas D, Stanton C, Ross RP (2022). Metataxonomic mapping of the microbial diversity of Irish and Eastern Mediterranean cheeses. Foods.

[36] Faust K, Raes J (2016). CoNet app: inference of biological association networks using Cytoscape. F1000Res.

[37] Shannon P, Markiel A, Ozier O (2003). Cytoscape: a software environment for integrated models of biomolecular interaction networks. Genome Res.

[38] Barbieri F, Tabanelli G, Montanari C (2021). Mediterranean spontaneously fermented sausages: spotlight on microbiological and quality features to exploit their bacterial biodiversity. Foods.

[39] Huang Z, Shen Y, Huang X, Qiao M, He RK, Song L (2021). Microbial diversity of representative traditional fermented sausages in different regions of China. J Appl Microbiol.

[40] Prado N, Sampayo M, González P, Lombó F, Díaz J (2019). Physicochemical, sensory and microbiological characterization of Asturian Chorizo, a traditional fermented sausage manufactured in Northern Spain. Meat Sci.

[41] Liu Y, Wan Z, Yohannes KW (2020). Functional characteristics of lactobacillus and yeast single starter cultures in the ripening process of dry fermented sausage. Front Microbiol.

[42] Drosinos EH, Paramithiotis S, Kolovos G, Tsikouras I, Metaxopoulos I (2007). Phenotypic and technological diversity of lactic acid bacteria and staphylococci isolated from traditionally fermented sausages in southern Greece. Food Microbiol.

[43] Montel M, Masson F, Talon R (1998). Bacterial role in flavour development. Meat Sci.

[44] Bhattacharya D, Nanda PK, Pateiro M, Lorenzo JM, Dhar P, Das AK (2022). Lactic acid bacteria and bacteriocins: novel biotechnological approach for biopreservation of meat and meat products. Microorganisms.

[45] Arief II, Jenie BSL, Suryati T, Ayuningtyas G, Fuziawan A (2014). Antimicrobial activity of bacteriocin from indigenous *Lactobacillus plantarum* 2C12 and its application on beef meatball as biopreservative. J Indonesian Trop Anim Agric.

[46] Gao Y, Li D, Liu X (2015). Effects of *Lactobacillus sakei* C2 and sakacin C2 individually or in combination on the growth of *Listeria monocytogenes*, chemical and odor changes of vacuum-packed sliced cooked ham. Food Control.

[47] Aspri M, Bozoudi D, Tsaltas D, Hill C, Papademas P (2017). Raw donkey milk as a source of Enterococcus diversity: assessment of their technological properties and safety characteristics. Food Control.

[48] Tidona F, Meucci A, Povolo M (2018). Applicability of *Lactococcus hircilactis* and *Lactococcus laudensis* as dairy cultures. Int J Food Microbiol.

[49] https://isappscience.org/wp-content/uploads/2019/04/probiotic_guidelines.pdf.

[50] Seleshe S, Kang SN (2021). Effect of different *Pediococcus pentosaceus* and *Lactobacillus plantarum* strains on quality characteristics of dry fermented sausage after completion of ripening period. Food Sci Anim Resour.

[51] Porto MC, Kuniyoshi TM, Azevedo PO, Vitolo M, Oliveira RP (2017). *Pediococcus* spp.: an important genus of lactic acid bacteria and pediocin producers. Biotechnol Adv.

[52] Ilavenil S, Vijayakumar M, Kim DH (2016). Assessment of probiotic, antifungal and cholesterol lowering properties of *Pediococcus pentosaceus* KCC-23 isolated from Italian ryegrass. J Sci Food Agric.

[53] Fugaban JII, Vazquez Bucheli JE, Park YJ (2022). Antimicrobial properties of *Pediococcus acidilactici* and *Pediococcus pentosaceus* isolated from silage. J Appl Microbiol.

[54] Kabiraz MP, Majumdar PR, Mahmud MMC, Bhowmik S, Ali A (2023). Conventional and advanced detection techniques of foodborne pathogens: a comprehensive review. Heliyon.

[55] Chang SH, Chen CH, Tsai GJ (2020). Effects of chitosan on *Clostridium perfringens* and application in the preservation of pork sausage. Mar Drugs.

[56] Pernu N, Keto-Timonen R, Lindström M, Korkeala H (2020). High prevalence of *Clostridium botulinum* in vegetarian sausages. Food Microbiol.

[57] Muratoglu K, Akkaya E, Hampikyan H, Bingol EB, Cetin O, Colak H (2020). Detection, characterization and antibiotic susceptibility of *Clostridioides* (*Clostridium*) *difficile* in meat products. Food Sci Anim Resour.

[58] Mladenović KG, Grujović MŽ, Kiš M (2021). Enterobacteriaceae in food safety with an emphasis on raw milk and meat. Appl Microbiol Biotechnol.

[59] Gwida M, Hotzel H, Geue L, Tomaso H (2014). Occurrence of *Enterobacteriaceae* in raw meat and in human samples from egyptian retail sellers. Int Sch Res Notices.

[60] Jansen W, Woudstra S, Müller A (2018). The safety and quality of pork and poultry meat imports for the common European market received at border inspection post Hamburg Harbour between 2014 and 2015. PLoS One.

[61] Nováková D, Sedláček I, Pantůček R, Štětina V, Švec P, Petráš P (2006). *Staphylococcus equorum* and *Staphylococcus succinus* isolated from human clinical specimens. J Med Microbiol.

[62] (2021). Prpich NZ, Camprubí GE, Cayré ME, Castro MP. Indigenous microbiota to leverage traditional dry sausage production. Int J Food Sci.

[63] Van Ba H, Seo H, Kim J (2016). The effects of starter culture types on the technological quality, lipid oxidation and biogenic amines in fermented sausages. LWT.

[64] (2013). EFSA Panel on Biological Hazards. Scientific opinion on the maintenance of the list of QPS biological agents intentionally added to food and feed (2013 update). EFSA J.

[65] Erkmen O https://www.researchgate.net/publication/354968527_Microbiological_Analysis_of_Foods_and_Food_Processing_Environments.

[66] Wen R, Sun F, Li XA, Chen Q, Kong B (2021). The potential correlations between the fungal communities and volatile compounds of traditional dry sausages from Northeast China. Food Microbiol.

[67] Ramos-Moreno L, Ruiz-Pérez F, Rodríguez-Castro E, Ramos J (2021). *Debaryomyces hansenii* is a real tool to improve a diversity of characteristics in sausages and dry-meat products. Microorganisms.

[68] Cano-García L, Rivera-Jiménez S, Belloch C, Flores M (2014). Generation of aroma compounds in a fermented sausage meat model system by *Debaryomyces hansenii* strains. Food Chem.

[69] Flores M, Corral S, Cano-García L, Salvador A, Belloch C (2015). Yeast strains as potential aroma enhancers in dry fermented sausages. Int J Food Microbiol.

[70] Prista C, Michán C, Miranda IM, Ramos J (2016). The halotolerant *Debaryomyces hansenii*, the Cinderella of non-conventional yeasts. Yeast.

[71] Angulo M, Reyes-Becerril M, Medina-Córdova N, Tovar-Ramírez D, Angulo C (2020). Probiotic and nutritional effects of *Debaryomyces hansenii* on animals. Appl Microbiol Biotechnol.

[72] Jain U, Ver Heul AM, Xiong S (2021). *Debaryomyces* is enriched in Crohn’s disease intestinal tissue and impairs healing in mice. Science.

[73] Haarer BK, Petzold A, Lillie SH, Brown SS (1994). Identification of MYO4, a second class V myosin gene in yeast. J Cell Sci.

[74] Makky EA, AlMatar M, Mahmood MH, Ting OW, Qi WZ (2021). Evaluation of the antioxidant and antimicrobial activities of ethyl acetate extract of *Saccharomyces cerevisiae*. Food Technol Biotechnol.

[75] Alarcón M, Pérez-Coello MS, Díaz-Maroto MC, Alañón ME, García-Ruiz A, Soriano A (2021). Inactive dry yeast to improve the oxidative stability of Spanish dry-fermented sausage “salchichón”. LWT.

[76] Kokkinofta R, Fotakis C, Zervou M (2017). Isotopic and elemental authenticity markers: a case study on cypriot wines. Food Anal Methods.

