# Supplementary material for: Regional microbial content of fermented traditional and industrial East Mediterranean sausages from the islands of Cyprus and Mytilini
Source: Microbiome Res Rep. 2024 Oct 28;4(1):3. doi: 10.20517/mrr.2024.47 (PMC11977371; doi:10.20517/mrr.2024.47)
Supplement: Supplementary file 1 [file mrr-4-1-3-SupplementaryMaterials.pdf]

Supplementary Materials

Regional microbial content of fermented traditional and industrial East Mediterranean sausages from the islands of Cyprus and Mytilini

Eleni Kamilari[1,2], Catherine Stanton[1,2,3], Dimitrios Tsaltas[4], R. Paul Ross[1,2]

[1]APC Microbiome Ireland, University College Cork, Cork T12 YT20, Ireland.
[2]School of Microbiology, University College Cork, Cork T12 K8AF, Ireland.
[3]Department of Biosciences, Teagasc Food Research Centre, Moorepark, Fermoy, Co., Cork P61 C996, Ireland.
[4]Department of Agricultural Sciences, Biotechnology and Food Science, Cyprus University of Technology, Lemesos 3036, Cyprus.

Correspondence to: Prof. R. Paul Ross, APC Microbiome Ireland, University College Cork, College Road, Cork T12 YT20, Ireland. E-mail: p.ross@ucc.ie

Supplementary Table 1. Sample information, bacterial alpha diversity indexes and observed OTUs

Sample ID	Reads passing filter	Denoised reads	Shannon	Simpson	Chao1	Observed OTUs
C1	34866	30328	3.40	0.81	60	59
C2	42065	35180	2.51	0.56	137	135
C3	53087	45522	2.02	0.50	51	49
C4	41463	35777	1.76	0.46	93	88
C5	47045	37810	5.31	0.95	199	196
C6	51424	44086	2.69	0.77	106	98
C7	40320	32457	3.87	0.80	397	381
C8	48691	42305	6.29	0.98	258	251
C9	52451	44820	3.39	0.86	52	52
C10	38348	33933	5.98	0.97	211	209
C11	44414	40334	1.28	0.42	33	32
C12	22895	19925	4.98	0.96	64	64
C13	44714	37743	3.12	0.68	103	102
C14	39443	34712	6.22	0.97	250	249
C15	39345	35574	2.58	0.78	40	39
C16	43365	37453	2.71	0.74	163	151
C17	30582	21594	5.93	0.90	696	696
C18	29865	26617	2.82	0.71	49	49
C19	39915	34975	3.49	0.82	73	70
C20	39395	35735	2.69	0.73	56	56
C21	34093	29832	3.57	0.82	129	123
C22	29202	26093	2.49	0.69	56	56
G1	49913	43062	1.40	0.31	64	63
G2	56576	46076	4.51	0.79	234	223
G3	55927	49410	1.16	0.25	73	71

G4	148347	107301	4.56	0.91	476	346
G5	41177	32277	4.43	0.90	217	212
G6	56933	45348	4.80	0.94	212	190
G7	36658	29695	3.63	0.84	185	181
G8	24538	19570	4.67	0.92	188	188
Sum	1357057.00	1135544.00	108.28	22.75	4925.00	4679.00
Average	45235.23	37851.47	3.61	0.76	164.17	155.97
Min	22895.00	19570.00	1.16	0.25	33.00	32.00
Max	148347.00	107301.00	6.29	0.98	696.00	696.00
STD	21487.04	15275.43	1.49	0.20	146.36	137.37

Supplementary Table 2. Sample information, fungal alpha diversity indexes and observed OTUs

Sample ID	Reads passing filter	Denoised reads	Shannon	Simpson	Chao1	Observed OTUs
C1	60498	30641	5.72	0.95	190	188
C2	72332	48657	5.85	0.93	271	261
C3	140726	73503	6.62	0.97	303	298
C4	74945	45733	6.28	0.96	262	259
C5	80979	44877	6.60	0.97	274	268
C6	59323	32650	6.35	0.97	233	230
C7	60234	46411	3.78	0.84	141	136
C8	83723	56999	5.30	0.90	320	296
C9	90902	81687	3.83	0.79	357	310
C10	42646	31056	2.91	0.74	57	57
C11	69182	41595	3.96	0.86	91	89
C12	56537	37212	2.48	0.67	56	56
C13	96059	63734	4.33	0.89	202	187
C14	57413	39516	3.75	0.78	159	157
C15	130279	60159	3.50	0.76	114	108
C18	101260	50614	5.35	0.89	283	271
C19	95328	47435	6.99	0.98	298	287
C20	92099	57955	5.21	0.93	221	216
C21	63571	55460	4.77	0.90	213	205
C22	67106	42160	5.56	0.96	192	187
G1	84725	46672	7.17	0.99	336	327
G4	43701	23571	6.78	0.98	209	208
G5	40395	38506	1.42	0.54	38	37
Sum	1763963	1096803	114.51	20.17	4820	4638
Average	76694	47687	5	1	210	202

Min	40395	23571	1	1	38	37
Max	140726	81687	7	1	357	327
STD	25575	13842	2	0	93	87

Supplementary Table 3. Comparison of the alpha microbial diversity of Cyprus and Mytilini sausages based on the Kruskal–Wallis test

Comparison	Microorganism	Group 1	Group 2	H	*P*-value	q-value
Country	Bacteria	Cyprus (n=22)	Greece (n=8)	0.092803	0.760643	0.760643
	Fungi	Cyprus (n=22)	Greece (n=3)	0.447552	0.5035	0.882206
Area	Bacteria	Limassol (n=2)	Mytilini (n=8)	0.5	0.4795	1
		Limassol (n=2)	Nicosia (n=13)	0.259615	0.610385	1
		Limassol (n=2)	Pitsilia (n=7)	0	1	1
		Mytilini (n=8)	Nicosia (n=13)	0.001115	0.973364	1
		Mytilini (n=8)	Pitsilia (n=7)	0.137255	0.711025	1
		Nicosia (n=13)	Pitsilia (n=7)	1.508634	0.219348	1
	Fungi	Limassol (n=2)	Mytilini (n=3)	0.333333	0.563703	0.654721
		Limassol (n=2)	Nicosia (n=13)	3.490385	0.061726	0.185179
		Limassol (n=2)	Pitsilia (n=5)	0.6	0.438578	0.654721
		Mytilini (n=3)	Nicosia (n=13)	0.366516	0.54491	0.654721
		Mytilini (n=3)	Pitsilia (n=5)	0.2	0.654721	0.654721
		Nicosia	Pitsilia	3.694737	0.054584	0.185179

		(n=13)	(n=5)			
Traditional	Bacteria	No (n=18)	Yes (n=12)	0.125	0.723674	0.723674
	Fungi	No (n=17)	Yes (n=6)	1.290179	0.256015	0.256015
Smoked	Bacteria	No (n=4)	Yes (n=26)	0.321429	0.57075	0.57075
	Fungi	No (n=5)	Yes (n=18)	0.2	0.654721	0.654721
Preservatives	Bacteria	No (n=15)	Yes (n=15)	1.139063	0.285851	0.285851
	Fungi	No (n=13)	Yes (n=10)	0.65	0.420113	0.420113

Supplementary Table 4. Comparison of microbial beta diversity in sausages based on production area and method (traditional or industrial), using the PERMANOVA pairwise test

Comparison	Microorganism	UniFrac metric	Group 1	Group 2	Sample size	Permutations	pseudo-F	*P*-value	q-value
Area	Bacteria	Weighted	Limassol	Mytilini	11	999	2.351625	0.058	0.348
			Limassol	Nicosia	15	999	1.618025	0.24	0.48
			Limassol	Pitsilia	9	999	0.48159	0.726	0.726
			Mytilini	Nicosia	22	999	1.922309	0.138	0.414
			Mytilini	Pitsilia	16	999	0.973037	0.475	0.6576
			Nicosia	Pitsilia	20	999	0.754822	0.548	0.6576
		Unweighted	Limassol	Mytilini	11	999	0.976916	0.493	0.5916
			Limassol	Nicosia	15	999	1.407474	0.098	0.294
			Limassol	Pitsilia	9	999	0.8546	0.697	0.697
			Mytilini	Nicosia	22	999	1.653585	0.02	0.12
			Mytilini	Pitsilia	16	999	0.970492	0.473	0.5916
			Nicosia	Pitsilia	20	999	1.30623	0.16	0.32
Traditional	Bacteria	Unweighted	No	Yes	22	999	0.74098	0.574	0.574
		Weighted	No	Yes	22	999	0.879597	0.603	0.603
	Fungi	Unweighted	No	Yes	20	999	1.214718	0.163	0.163
		Weighted	No	Yes	20	999	1.673776	0.144	0.144
